# Initial Alignment Algorithm Based on the DMCS Method in Single-Axis RSINS with Large Azimuth Misalignment Angles for Submarines

**DOI:** 10.3390/s18072123

**Published:** 2018-07-02

**Authors:** Xiu-Wei Xia, Qian Sun

**Affiliations:** 1 College of Automation, Harbin Engineering University, Harbin 150001, China; xiaxw2012@yeah.net; 2 College of Information and Communication Engineering, Harbin Engineering University, Harbin 150001, China

**Keywords:** initial alignment, Rotation Strapdown Inertial Navigation System (RSINS), submarine, Dual Mathematical Calculation System (DMCS), large misalignment angle

## Abstract

Since the inertial sensor error has been modulated effectively by the Rotation Modulation Technique (RMT), the Rotation Strapdown Inertial Navigation System (RSINS) has been widely used for submarines in order to satisfy the requirement of high-accuracy and long working duration. The performance of the initial alignment is main factor affecting the accuracy of the Strapdown Inertial Navigation System (SINS). The traditional initial alignment algorithm based on the compass method has bad performance when the misalignment angle is large, which will make the submarine SINS fail to launch properly in a complex operating environment. Since the RSINS uses the mathematical platform to calculate the navigation information, it allows multiple algorithms to run simultaneously, and different algorithms do not interact with each other. Thus, to improve the alignment accuracy, an initial alignment algorithm based on the Dual Mathematical Calculation System (DMCS) is proposed; moreover, to solve the problem of large azimuth misalignment angle, an improved DMCS-based alignment algorithm is also presented in this paper. Both simulations and experiments showed that the novel algorithm can effectively improve the initial alignment performance under the large misalignment angle environment, enhancing the environmental suitability of the RSINS.

## 1. Introduction

The Strapdown Inertial Navigation System (SINS) can provide the attitude, velocity and position of a vehicle by utilizing the gyroscope and the accelerometer to measure its linear velocity and angular rate, respectively. Due to its numerous merits, such as being autonomous, all-weather, strong real time, multi-parameter measurement, and so on, SINS has been widely used in submarines and other underwater vehicles [1,2,3]. However, the accumulated positioning error caused by the inertial sensor error will severely affect the navigation performance of underwater vehicles [4,5]. In addition, with the rapid development of underwater navigation technology, the requirement of SINS performance is much higher and higher. Therefore, to improve the SINS’ performance in long-term navigation systems, reducing and compensating the inertial sensor error are becoming critical problems now.

Through the periodic rotation of the turntable, the inertial sensor error can be modulated by the Rotation Modulation Technique (RMT) into the sine or cosine signal, the integral of which is zero in an entire period, thereby restraining the influence of the inertia sensor error [6,7]. Without any external information, the RMT is an effective self-compensation technique, so it has been widely used in error suppression of the SINS. According to the number of rotational axes, the RMT is divided into three kinds, including the single-axis RMT, the dual-axis RMT and the tri-axis RMT [8,9]. Compared with other RMT, although the single-axis RMT can only modulate the constant inertial sensor error along with the vertical direction of the rotation axis, it is simpler and easier to operate. Hence, it is more widely used in the practical application of inertial navigation [10,11,12,13].

A self-compensation method based on single-axis RMT was proposed in [10,14] to inhibit the constant deviation of inertial sensors in the Rotation Strapdown Inertial Navigation System (RSINS), and the navigation accuracy improved significantly compared with traditional navigation systems. However, the initial alignment error is still a factor that restricts the further improvement of system accuracy. The same as the traditional SINS, the initial alignment was carried out firstly to determine the initial attitude of the single-axis RSINS according to the inertial sensors’ information [13,15,16]. The initial alignment error in practical will always exist in the output of RSINS and seriously affects its navigation accuracy. Therefore, the initial alignment is of great importance to enhance the performance of RSINS.

Depending on whether using external information or not, the initial alignment is divided into the self-alignment and the integrated alignment. Due to its autonomy, the self-alignment method is one of the most commonly-used methods in submarine navigation systems.The self-alignment process is generally divided into two stages, the coarse alignment and the fine alignment. In the coarse alignment, the gravity acceleration and angular rate of the Earth’s rotation measured by accelerometers and gyroscopes are used to calculate the vehicle’s initial attitude coarsely. On this basis, the fine alignment uses the compass effect to further calculate the vehicle’s accurate attitude [17,18]. The error of the coarse alignment is generally less than one degree, while the one of the fine alignment is smaller, approximately several arcminutes [19,20,21]. Furthermore, the performance of the coarse alignment directly affects the performance of the fine alignment. The accuracy and the convergence speed are two main criteria for evaluating alignment methods.

A fast alignment method based on the open-loop mathematical platform misalignment model in single-axis RSINS was proposed in [2]. The RMT was adopted to reduce the influence of inertial sensors’ errors, and the Kalman filter was employed to suppress the effect of the environmental disturbance on the initial alignment accuracy in [22,23]. In the above references, misalignment angles were assumed as small angles, and then, the system can be approximated as a linear system. However, the misalignment angle obtained from the coarse alignment actually cannot meet the requirement of the fine alignment due to the vehicle maneuvering and the external disturbance or pressure caused by the complex underwater environment. As a result, the azimuth misalignment angle is usually large, and a large amount of nonlinear errors will be introduced if the system is still approximately linear [24,25]. Besides, the performance of the initial alignment usually deteriorates in this condition. Although nonlinear filters, such as the Extended Kalman Filter (EKF), the Unscented Kalman Filter (UKF) or the Particle Filter (PF), can solve the nonlinear problem, the system modeling and the computation are both complex in actual systems. Therefore, other ways to solve the nonlinear problem of the actual navigation system are needed.

Unlike the platform-based inertial navigation system, the SINS and the RSINS both use a mathematics platform to calculate the inertial navigation information, and the data collected by inertial sensors can truly reflect the vehicle’s motion [3,26]. Therefore, the RSINS allows multiple algorithms to run simultaneously, and the algorithms do not affect each other. Thus, based on this, an alignment method based on the Dual Mathematical Calculation System (DMCS) can be realized [6].

In order to improve the performance of the RSINS for submarines, firstly a novel initial alignment algorithm based on the DMCS and traditional compass self-aligning method is presented in this paper. The alignment accuracy can be improved by utilizing the DMCS. Moreover, an improved DMCS-based initial alignment algorithm is proposed to further eliminate the influence of the nonlinear error caused by the large azimuth misalignment angle. Though the reasonable design of the control variable and the system parameters, the RSINS can still be approximated as a linear model without introducing any nonlinear errors with this improved algorithm, suppressing the influence of nonlinear errors on the alignment speed and enhancing the performance of RSINS’ initial alignment availably.

The paper is organized as follows. Section 2 introduces the principles of the single-axis RSINS and the initial alignment of the RSINS. A novel alignment algorithm based on the DMCS and traditional compass self-aligning method and an improved DMCS-based initial alignment algorithm with a large azimuth misalignment angle of the RSINS for submarines are proposed in Section 3. Section 4 and Section 5 give the simulations and real tests, respectively. Section 6 concludes the whole manuscript.

## 2. Basic Knowledge

### 2.1. The Principle of Single-Axis RSINS

In the RSINS, the Inertial Measurement Unit (IMU) is installed on a turntable, which is mounted on the vehicle. The IMU is rotated according to a certain rule by controlling the turntable, and thus, the inertial sensor error can be modulated as a periodic signal, which can be eliminated during an integration period, reducing the influence of the inertial sensor on the navigation accuracy. The navigation solution of the RSINS is similar to the one of the traditional SINS, except that the output attitude should be decoupled according to the rotation information given by the turntable [24]. Therefore, in this manuscript, to improve the accuracy of the submarine’s navigation system, the RSINS based on the RMT is used.

**Figure 1:**
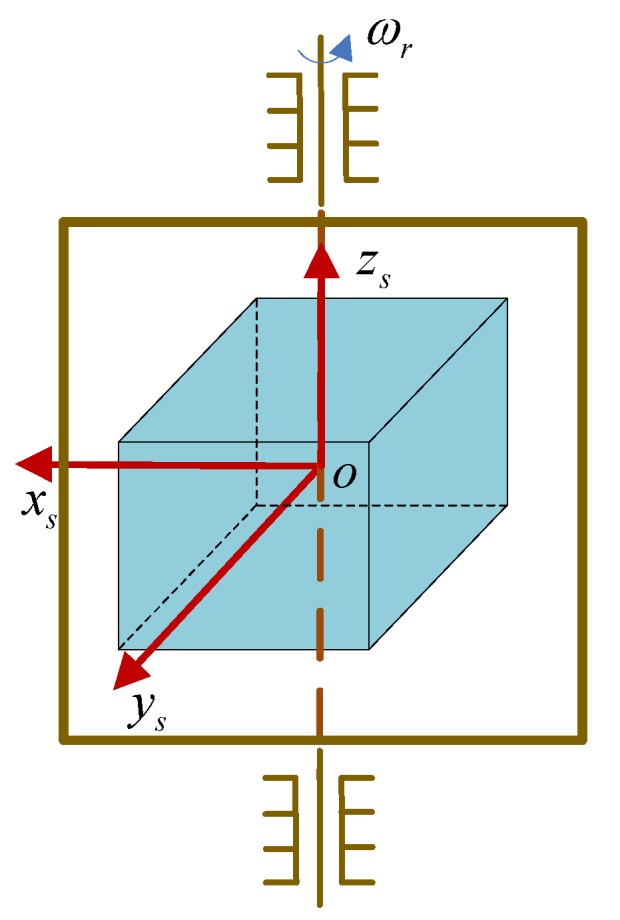
Installation diagram of the single-axis RSINS.

The installation diagram of single-axis RSINS is illustrated in Figure 1. The $ox_{s}y_{s}z_{s}$ coordinate frame, denoted by *s*, is the body coordinate frame that rotates with the single rotation axis; *b* is the body coordinate frame actually used in the RSINS. Assume that *s* and *b* are coincident when the single-axis turntable is at the zero position, and in this installation mode, the relationship between the measurement in *b* and *s* is shown as follows:

(1) $$\left\{\begin{array}{c} f^{b}=C_{s}^{b}f^{s}\\ \omega_{ib}^{b}=C_{s}^{b}\omega_{is}^{s}+\omega_{sb}^{b} \end{array}\right.$$

where $f^{b}$, $f^{s}$ are the specific force in the *b* frame and *s* frame; $\omega_{ib}^{b}$, $\omega_{is}^{s}$ denote the angular rate in the *b* frame and *s* frame, respectively; $\omega_{sb}^{b}$ is the projection of the rotation angular rate of the *b* frame to *s* frame in the *b* frame; $C_{s}^{b}$ indicates the coordinate transformation matrix between the *s* frame and *b* frame. Since the rotation angular rate of the turntable $\omega_{r}$ is known, $C_{s}^{b}$ can be described as:

(2) $$C_{s}^{b}=\left[\begin{array}{ccc} \cos\omega_{r}t & -\sin\omega_{r}t & 0\\ \sin\omega_{r}t & \cos\omega_{r}t & 0\\ 0 & 0 & 1 \end{array}\right]$$

(3) $$\omega_{sb}^{b}={\left[\begin{array}{ccc} 0 & 0 & -\omega_{r} \end{array}\right]}^{⊤}$$

where *t* is the rotation time.

Define that ${\tilde{f}}^{s}$ and ${\tilde{\omega }}_{is}^{s}$ are inertial sensor measurements:

(4) $$\left\{\begin{array}{c} {\tilde{f}}^{s}=C_{b}^{s}f^{b}+\nabla^{s}\\ {\tilde{\omega }}_{is}^{s}=C_{b}^{s}\omega_{ib}^{b}-\omega_{sb}^{b}+\varepsilon^{s} \end{array}\right.$$

wherein $\nabla^{s}={\left[\begin{array}{ccc} \nabla_{sx} & \nabla_{sy} & \nabla_{sz} \end{array}\right]}^{⊤}$, $\varepsilon^{s}={\left[\begin{array}{ccc} \varepsilon_{sx} & \varepsilon_{sy} & \varepsilon_{sz} \end{array}\right]}^{⊤}$ are the constant accelerometer bias and the constant gyroscope drift, respectively.

In the case that $C_{s}^{b}$ and $\omega_{sb}^{b}$ are already known, we can obtain the measurement in *b*, denoted by ${\tilde{f}}^{b}$ and ${\tilde{\omega }}_{ib}^{b}$:

(5) $$\left\{\begin{array}{c} {\tilde{f}}^{b}=C_{s}^{b}{\tilde{f}}^{s}=f^{b}+C_{s}^{b}\nabla^{s}\\ {\tilde{\omega }}_{ib}^{b}=C_{s}^{b}{\tilde{\omega }}_{is}^{s}+\omega_{sb}^{b}=\omega_{ib}^{b}+C_{s}^{b}\varepsilon^{s} \end{array}\right.$$

Therefore, the corresponding sensor error is shown as follows:

(6) $$\nabla^{b}=C_{s}^{b}\nabla^{s}=\left[\begin{array}{c} \nabla_{sx}\cos\omega_{r}t-\nabla_{sy}\sin\omega_{r}t\\ \nabla_{sx}\sin\omega_{r}t+\nabla_{sy}\cos\omega_{r}t\\ \nabla_{sz} \end{array}\right]$$

(7) $$\varepsilon^{b}=C_{s}^{b}\varepsilon^{s}=\left[\begin{array}{c} \varepsilon_{sx}\cos\omega_{r}t-\varepsilon_{sy}\sin\omega_{r}t\\ \varepsilon_{sx}\sin\omega_{r}t+\varepsilon_{sy}\cos\omega_{r}t\\ \varepsilon_{sz} \end{array}\right]$$

From Equations (6) and (7), we can see that sensor errors in the *x*-axis and *y*-axis are modulated as periodic signals, whose integrals are zeros throughout the period theoretically. After setting an appropriate rotating angular rate $\omega_{r}$ utilizing the method presented in [27], this kind of periodic signal can be eliminated to some extent. Therefore, the single-axis RSINS can effectively reduce the influence of the inertial sensor error on the navigation accuracy, thereby improving the navigation accuracy.

### 2.2. The Initial Alignment in Single-Axis RSINS

#### 2.2.1. Coarse Alignment

Since the RSINS’s working principle is similar to the traditional SINS, except the attitude decoupling, the coarse alignment of RSINS is also divided into the horizontal coarse alignment and the azimuth coarse alignment, which are usually carried out at the same time.

The horizontal coarse alignment is performed by utilizing the gravity vector, which is used to calculate the horizontal attitude angle of vehicles in the current moment roughly. To simplify the analysis without loss of generality, we assume that the body frame *b* coincides with the navigation coordinate frame (*n*), that means $C_{b}^{n}=I$, and then, the accelerometer bias and the gyroscope drift in *n* frame after modulation are:

(8) $$\left\{\begin{array}{c} \Delta A_{x}=\nabla_{sx}\cos\omega_{r}t-\nabla_{sy}\sin\omega_{r}t\\ \Delta A_{y}=\nabla_{sx}\sin\omega_{r}t+\nabla_{sy}\cos\omega_{r}t\\ \Delta A_{z}=\nabla_{sz} \end{array}\right.$$

(9) $$\left\{\begin{array}{c} \varepsilon_{x}=\varepsilon_{sx}\cos\omega_{r}t-\varepsilon_{sy}\sin\omega_{r}t\\ \varepsilon_{y}=\varepsilon_{sx}\sin\omega_{r}t+\varepsilon_{sy}\cos\omega_{r}t\\ \varepsilon_{z}=\varepsilon_{sz}\cos\omega_{r}t-\varepsilon_{sy}\sin\omega_{r}t \end{array}\right.$$

where in the variable $V_{x,y,z}$ means the component of the variable *V* in the *n* frame along with the *x*-axis, *y*-axis and *z*-axis.

Therefore, the horizontal components of the gravity acceleration measured by accelerometers and gyroscopes are shown as follows:

(10) $$\left\{\begin{array}{c} \Delta A_{x}=-\beta g\\ \Delta A_{y}=\alpha g \end{array}\right.$$

where *g* is the gravity acceleration; α, β are the north and east horizontal misalignment angles, respectively. In horizontal coarse alignment, the initial horizontal attitude of vehicles can be roughly calculated by using Equation (8) in a short time.

The azimuth rough alignment uses the projection of the Earth’s rotation angular rate in the local north direction to roughly calculate the azimuth attitude angle of vehicles at the current moment, shown as (9).

(11) $$\varepsilon_{x}=\gamma \omega_{ie}\cos L$$

where in *L* is the local latitude, $\omega_{ie}$ denotes the Earth’s rotation angular rate and γ presents the azimuth misalignment angle.

Therefore, in the coarse alignment, initial attitude angles of the vehicle can be calculated roughly and quickly by utilizing the gravitational acceleration and the Earth’s rotation angular rate [23].

#### 2.2.2. Fine Alignment

Based on the coarse alignment, the fine alignment is proceeded. Compared with the coarse alignment, the fine alignment time is longer, and the accuracy is higher. Aimed at the traditional SINS or RSINS for ships and submarines, the fine alignment is usually done in about thirty minutes, while the coarse alignment is done in two minutes. The level of the fine alignment accuracy can be decreased from degrees to arcmin. Similarly, the fine alignment is also divided into the horizontal fine alignment and the azimuth fine alignment. The former will be proceeded firstly, and then, both of them proceed at the same time [3,23].

In the horizontal fine alignment, the difference between the gravity acceleration and the actual measured acceleration is used. Acceleration errors and misalignment angles in north and east horizontal loops are shown as:

(12) $$\left\{\begin{array}{c} \delta {\dot{V}}_{y}=-2\omega_{ie}\sin L\delta V_{x}+\alpha g+\Delta A_{y}\\ \dot{\alpha }=\frac{\delta V_{y}}{R}+\omega_{ie}\beta \sin L-\omega_{ie}\gamma \cos L+\varepsilon_{x} \end{array}\right.$$

(13) $$\left\{\begin{array}{c} \delta {\dot{V}}_{x}=2\omega_{ie}\sin L\cdot \delta V_{y}-\beta g+\Delta A_{x}\\ \dot{\beta }=\frac{\delta V_{x}}{R}-\omega_{ie}\alpha \sin L+\varepsilon_{y} \end{array}\right.$$

where $\delta V_{x}$, $\delta V_{y}$ are the vehicle’s velocity errors, respectively; and *R* is the radius of the Earth.

**Figure 2:**
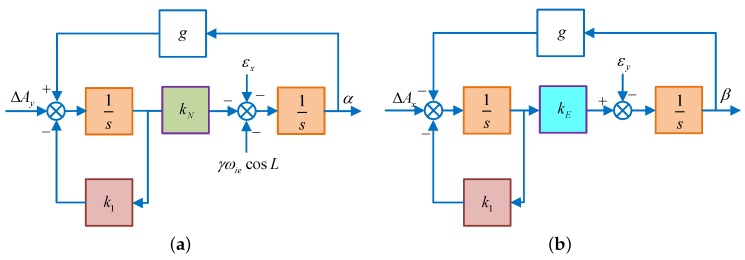
The schematic diagram of horizontal fine alignment. (**a**) Schematic diagram of horizontal fine alignment in the north loop; (**b**) schematic diagram of horizontal fine alignment in the east loop.

As we all known that in a pure SINS, the system is in a critical stable state and its errors are periodicity oscillating with the Schuler oscillation frequency [8,25], it is necessary to exert control over the system to ensure its stability. Introducing the damping in the SINS horizontal loop and changing the integral part into the inertial part of the acceleration, then the system will work in the damping state [1,17,28]. To further shorten the oscillation period, the proportional parameter 1/R is replaced by $k_{N}$ and $k_{E}$ in the north and east loops, receptively. Diagrams of the horizontal fine alignment are shown in Figure 2.

According to the Mason gain formula, the characteristic polynomial of the RSINS can be obtained as:

(14) $$\left\{\begin{array}{c} \Delta_{N}\left(s\right)=s^{2}+k_{1}s+k_{N}\omega_{s}^{2}\\ \Delta_{E}\left(s\right)=s^{2}+k_{1}s+k_{E}\omega_{s}^{2} \end{array}\right.$$

According to the final value theorem, the steady-state error of the RSINS can be obtained as:

(15) $$\left\{\begin{array}{c} \alpha_{s}=-\frac{1}{g}\cdot \Delta A_{y}-\frac{k_{1}}{k_{N}g}\cdot \left(\varepsilon_{x}+\gamma \omega_{ie}\cos L\right)\\ \beta_{s}=\frac{1}{g}\cdot \Delta A_{x}-\frac{k_{1}}{k_{E}g}\cdot \varepsilon_{y} \end{array}\right.$$

**Figure 3:**
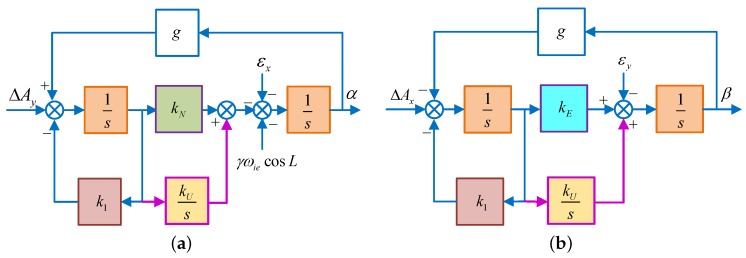
The schematic diagram of horizontal fine alignment. (**a**) Schematic diagram of horizontal fine alignment in the north loop; (**b**) schematic diagram of horizontal fine alignment in the east loop.

In addition, to eliminate the influence of the gyro bias and azimuth misalignment angle on the steady accuracy of the horizontal alignment, an integration link $k_{U}/s$ is introduced to calculate the control angle rate. This is as shown in Figure 3.

According to the Mason gain formula, the characteristic polynomial of the RSINS can be obtained as:

(16) $$\left\{\begin{array}{c} \Delta_{N}\left(s\right)=s^{3}+k_{1}s^{2}+k_{N}gs+k_{U}g\\ \Delta_{E}\left(s\right)=s^{3}+k_{1}s^{2}+k_{E}gs+k_{U}g \end{array}\right.$$

Now, the system is still asymptotically stable, and the steady-state error can be expressed as:

(17) $$\left\{\begin{array}{c} \alpha_{s}=-\frac{1}{g}\cdot \Delta A_{y}\\ \beta_{s}=\frac{1}{g}\cdot \Delta A_{x} \end{array}\right.$$

**Figure 4:**
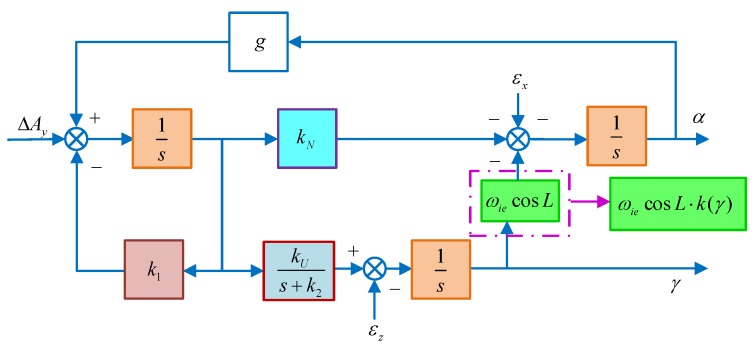
The diagram of the gyrocompass alignment in the north loop.

The azimuth alignment is performed on the basis of the horizontal alignment, aimed at the SINS mathematical platform being northward and determining the initial azimuth angle. In traditional initial alignment methods, the compass alignment method is the most commonly-used azimuth alignment, using the compass effect to adjust the azimuth misalignment angle. The coupling relationship between the azimuth error and the north horizontal loop is called the compass effect, so this kind of initial alignment, the horizontal alignment plus the azimuth compass alignment, is usually named as the compass alignment method directly. Assuming that the azimuth misalignment angle is small, which means, k(γ)=sinγ/γ, γ≈0, sinγ≈γ, k(γ)≈1, the diagram of the traditional compass alignment method is shown in Figure 4.

According to the Mason gain formula, the characteristic polynomial of the system can be obtained as:

(18) $$\Delta_{U}\left(s\right)=s^{4}+\left(1+k_{2}\right)s^{3}+\left(k_{1}k_{2}+k_{N}R\omega_{s}^{2}\right)s^{2}+k_{N}k_{2}R\omega_{s}^{2}s+k_{U}R\omega_{ie}\cos L\omega_{s}^{2}$$

The system is still asymptotically stable system, and the steady-state error can be expressed as:

(19) $$\gamma_{s}=\frac{k_{2}k_{N}R}{k_{U}R\omega_{ie}\cos L}\varepsilon_{z}+\frac{1}{\omega_{ie}\cos L}\varepsilon_{x}$$

From Equations (15), (17) and (19), we can see that horizontal components of inertial sensor errors are modulated to periodic signals, while the vertical component is not. Equation (19) presents that the steady-state error of the azimuth misalignment is related to $\varepsilon_{z}$, which cannot be modulated by the single-axis RMT. Thus, in single-axis RSINS, the accuracy of azimuth misalignment angle cannot be significantly improved, different from the ones of horizontal misalignment angles. Furthermore, if the azimuth misalignment is large, nonlinear terms will appear in the traditional compass alignment method, seriously affecting the accuracy of the initial alignment.

## 3. A Novel DMCS-Based Initial Alignment Algorithm with a Large Azimuth Misalignment Angle

### 3.1. Initial Alignment Algorithm Based on the DMCS

Comparing Equation (15) with (17), their difference contains the azimuth information, expressed as:

(20) $$\alpha_{s}^{1}-\alpha_{s}^{2}=-\frac{k_{1}}{k_{N}g}\cdot \left(\varepsilon_{x}+\gamma \omega_{ie}\cos L\right)$$

where $\alpha_{s}^{1}$, $\alpha_{s}^{2}$ denote the north horizontal misalignment angle of the RSINS, which is illustrated in Figure 2a and Figure 3a, respectively. If ignoring the east gyroscope drift, the azimuth misalignment angle can be obtained by Equation (20). Therefore, after the coarse alignment of the RSINS, control methods, illustrated in Figure 2 and Figure 3, are used to proceed with the horizontal alignment, respectively. When these two sets of alignment systems are stabilized, the azimuthal alignment can be achieved by Equation (20), achieving the initial alignment of the RSINS.

On the basis of the above theory, a novel initial alignment algorithm based on the DMCS is proposed in this manuscript. In this alignment algorithm, after completing the coarse alignment, two control modes are executed at the same time in the horizontal fine alignment, and then, the azimuth alignment can be achieved by obtaining the azimuth information from these two horizontal misalignment angles. Besides, the better result obtained by using the second control model shown in Figure 3 is taken as the final one. In this algorithm, the horizontal fine alignment shown as Figure 2 is named “System 1”, while the one shown as Figure 3 is named “System 2”.

**Figure 5:**
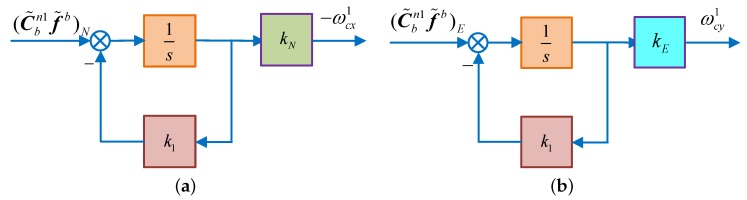
The schematic diagram of control angular rate in System 1. (**a**) Schematic diagram of calculating $\omega_{cx}^{1}$; (**b**) schematic diagram of calculating $\omega_{cy}^{1}$.

Since System 1 and System 2 share the same coarse alignment result, the initial misalignment angles of these two systems are the same. According to the above analysis, we can see that the focus of this proposed alignment algorithm is the calculation of control information and parameters. Taking System 1 as an example, the control angular rate added to the calculation of the RSINS attitude matrix is denoted by $\omega_{c}^{n1}$:

(21) $$\omega_{c}^{n1}={\left[\begin{array}{ccc} \omega_{cx}^{1} & \omega_{cy}^{1} & 0 \end{array}\right]}^{⊤}$$

where $\omega_{cx}^{1}$, $\omega_{cy}^{1}$ are calculated as Figure 5.

In Figure 5, ${\tilde{C}}_{b}^{n1}$ is the strapdown attitude matrix calculated by using System 1, and ${\tilde{f}}^{b}$ is the measured acceleration.

In order to ensure the stability, the parameters of System 1 are:

(22) k1=2ξωnkN=kE=ωn2ωn2gg

where ξ is the damping coefficient and $\omega_{n}$ is the natural oscillation frequency.

**Figure 6:**
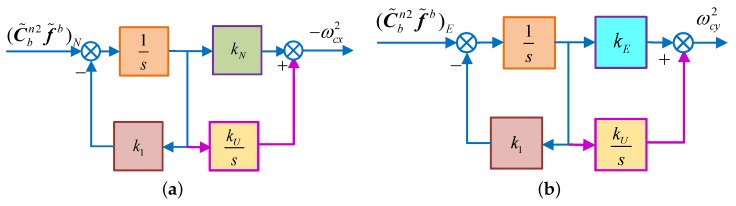
The schematic diagram of the control angular rate in System 2. (**a**) Schematic diagram of calculating $\omega_{cx}^{2}$; (**b**) schematic diagram of calculating $\omega_{cy}^{2}$.

Similarly, the control angular rate of System 2 is denoted by $\omega_{c}^{n2}$, and it can be calculated as Figure 6.

Here, ${\tilde{C}}_{b}^{n2}$ is the strapdown attitude matrix calculated in System 2. The parameters are set:

(23) k1=3ξωnkN=kE=ωn2(1+2ξ2)ωn2(1+2ξ2)ggkU=ξωn3ξωn3gg

In addition, due to the lack of a control angular rate in the azimuth during the horizontal alignment, the change of the azimuth misalignment angle is:

(24) $$\dot{\gamma }=-\omega_{ie}\cos L\cdot \alpha -\varepsilon_{z}$$

Since $\omega_{ie}\cos L\cdot \alpha$ and $\varepsilon_{z}$ are both very small, γ can be approximated as a constant during the whole alignment process. That is, the azimuthal misalignment angles of System 1 and System 2 are the same. Assuming that $\phi^{n1}$ is the misalignment angle between the calculated strapdown attitude matrix ${\tilde{C}}_{b}^{n1}$ and the true strapdown attitude matrix ${\tilde{C}}_{b}^{n}$ while $\phi^{n2}$ is the misalignment angle between ${\tilde{C}}_{b}^{n2}$ and ${\tilde{C}}_{b}^{n}$. The following equation holds:

(25) $$\left\{\begin{array}{c} {\tilde{C}}_{b}^{n1}=\left(I-\phi^{n1}\times \right){\tilde{C}}_{b}^{n}\\ {\tilde{C}}_{b}^{n2}=\left(I-\phi^{n2}\times \right){\tilde{C}}_{b}^{n} \end{array}\right.$$

From Equation (25), we can obtain:

(26) $$\left(\phi^{n1}-\phi^{n2}\right)\times =I-{\tilde{C}}_{b}^{n1}{\left[{\tilde{C}}_{b}^{n2}\right]}^{⊤}$$

where $\phi^{ni}\left(i=1,2\right)$ is the shew-symmetric matrix:

(27) $$\phi^{ni}=\left[\begin{array}{ccc} 0 & -\gamma^{i} & \beta^{i}\\ \gamma^{i} & 0 & -\alpha^{i}\\ -\beta^{i} & \alpha^{i} & 0 \end{array}\right]$$

Superscripts 1 and 2 in the above equation represent the relevant parameters of System 1 and System 2, respectively.

Assuming that $\Delta \alpha =\alpha^{1}-\alpha^{2}$, when System 1 and System 2 are both stable, the following equation is satisfied:

(28) $$\gamma =-\frac{\omega_{n}}{2\xi \omega_{ie}\cos L}\cdot \Delta \alpha_{s}-\frac{\varepsilon_{x}}{\omega_{ie}\cos L}$$

Define that $\hat{\gamma }$ is the estimation of γ, and:

(29) $$\hat{\gamma }=-\frac{\omega_{n}}{2\xi \omega_{ie}\cos L}\cdot \Delta \alpha$$

Thus, the azimuth misalignment angle can be estimated in real time by utilizing Equations (26) and (29), and the estimated error of the stable system is:

(30) $$\delta \gamma =-\frac{\varepsilon_{x}}{\omega_{ie}\cos L}$$

In order to ensure the stability of the azimuth alignment loop shown in Figure 4, the parameters in Figure 4 should be set as:

(31) k1=k2=2ξωnkN=ωn2(1+ξ2)ωn2(1+ξ2)ggkU=ξ2ωn4ξ2ωn4gωiecosLgωiecosL

**Figure 7:**
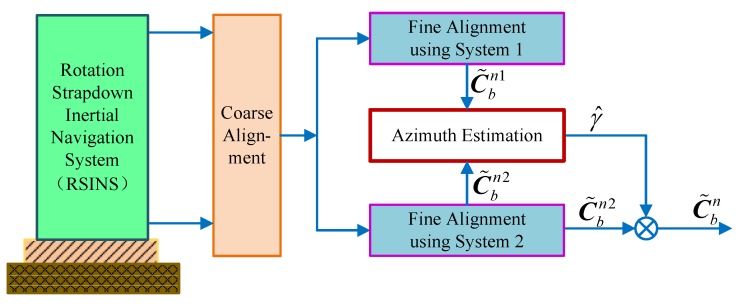
The schematic diagram of the DMCS-based alignment algorithm.

Therefore, we can realize the novel alignment algorithm based on the DMCS, shown as Figure 7. After completing the coarse alignment, the dual systems are executed simultaneously, calculating the strapdown attitude matrix ${\tilde{C}}_{b}^{n1}$ and ${\tilde{C}}_{b}^{n2}$. Then, the azimuth misalignment angle can be estimated by Equations (26) and (29). Afterwards, the corrected attitude matrix ${\tilde{C}}_{b}^{n}$ can be obtained by compensating ${\tilde{C}}_{b}^{n2}$, achieving the fine alignment of the RSINS.

Through the above theoretical analysis, we can see that this novel DMCS-based initial alignment algorithm of RSINS can not only eliminate the influence of the vertical gyroscope drift, but also improve the alignment accuracy availably. However, in the above analysis, the azimuth misalignment angle is ideally assumed to be a small one. However, it is extremely difficult to guarantee in actual systems, especially when the vehicle is violently maneuvering. Therefore, the presented DMCS-based alignment algorithm with a large azimuth misalignment angle should be discussed further.

### 3.2. Improved DMCS-Based Alignment Algorithm with a Large Misalignment Angle

**Figure 8:**
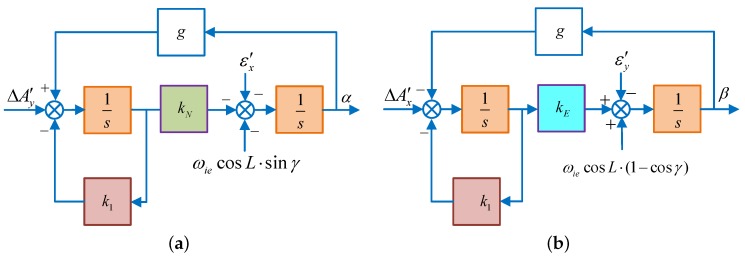
The schematic diagram of horizontal fine alignment in System 1. (**a**) Schematic diagram of horizontal fine alignment in the north loop; (**b**) schematic diagram of horizontal fine alignment in the east loop.

When the azimuth misalignment angle is large, the approximation, γ≈0, sinγ≈γ and cosγ≈1, is no longer applicable. Correspondingly, the control block diagram will change to Figure 8.

In Figure 8a,b, $\Delta A_{y}^{\prime }$, $\varepsilon_{x}^{\prime }$, $\Delta A_{x}^{\prime }$, $\varepsilon_{y}^{\prime }$ are equivalent senor errors shown as Equations (8) and (9) without the approximation.

According to the final value theorem, under large azimuth misalignment, the steady state value of the horizontal misalignment angle in System 1 is:

(32) $$\left\{\begin{array}{c} \alpha_{s}=-\frac{1}{g}\cdot \Delta A_{y}^{\prime }-\frac{k_{1}}{k_{N}g}\cdot \left({\varepsilon^{\prime }}_{x}+\omega_{ie}\cos L\cdot \sin\gamma \right)\\ \beta_{s}=\frac{1}{g}\cdot \Delta A_{x}^{\prime }-\frac{k_{1}}{k_{E}g}\cdot \left[\varepsilon_{y}^{\prime }+\left(\cos\gamma -1\right)\cdot \omega_{ie}\cos L\right] \end{array}\right.$$

Besides, when the azimuth misalignment angle is large, the $\omega_{ie}\cos L$ evolves from linear to nonlinear, indicated by $\omega_{ie}\cos L\cdot k\left(\gamma \right)$. In this case, $k_{U}$ should be set as:

(33) kU=ξ2ωn4ξ2ωn4[gωiecosL·k(γ)[gωiecosL·k(γ)]

**Figure 9:**
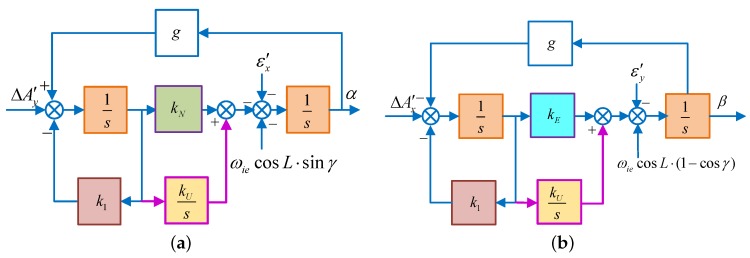
The schematic diagram of horizontal fine alignment in System 2. (**a**) Schematic diagram of horizontal fine alignment in the north loop; (**b**) schematic diagram of horizontal fine alignment in the east loop.

Similarly, the control block diagram of System 2 also changes into Figure 9:

According to the final value theorem, with large misalignment angle, the steady-state value of the horizontal misalignment angle in System 2 is the same as Equation (17), only the sensor error is not approximated. However, the parameters are usually set as Equation (31) due to the unknown k(γ), resulting in non-optimal pole configuration. With the increase of γ, the gap between $k_{U}$ and $k_{U}^{*}$ is gradually increased, this reducing the alignment speed of the azimuth alignment.

Moreover, the error source of the horizontal fine alignment is changed when the misalignment angle is large, and the proposed DMCS-based alignment algorithm described in Section 3.1 is no longer applicable. Now, we will improve this alignment algorithm in this part.

Assume that:

(34) $$\left\{\begin{array}{c} \Delta \alpha =\alpha^{1}-\alpha^{2}\\ \Delta \beta =\beta^{1}-\beta^{2} \end{array}\right.$$

When System 1 and System 2 are both stable, we can obtain:

(35) $$\left\{\begin{array}{c} \sin\gamma =-\frac{\omega_{n}}{2\xi \omega_{ie}\cos L}\cdot \Delta \alpha_{s}-\frac{{\varepsilon^{\prime }}_{x}}{\omega_{ie}\cos L}\\ \cos\gamma =1-\frac{\omega_{n}}{2\xi \omega_{ie}\cos L}\cdot \Delta \beta_{s}-\frac{{\varepsilon^{\prime }}_{y}}{\omega_{ie}\cos L} \end{array}\right.$$

Define that $\hat{s}$ and $\hat{c}$ are estimated values of sinγ and cosγ, respectively:

(36) $$\left\{\begin{array}{c} \hat{s}=-\frac{\omega_{n}}{2\xi \omega_{ie}\cos L}\cdot \Delta \alpha \\ \hat{c}=1-\frac{\omega_{n}}{2\xi \omega_{ie}\cos L}\cdot \Delta \beta \end{array}\right.$$

Therefore, the sine and cosine of the azimuth misalignment angle can be estimated by Equations (26) and (36). On this basis, ${\tilde{C}}_{b}^{n2}$ can be open-loop compensated, getting the corrected attitude matrix ${\tilde{C}}_{b}^{n}$ and achieving fine alignment with a large azimuth misalignment.

According to Mason’s gain formula, the frequency domain equation of $\hat{s}$ can be obtained according to Mason’s gain formula:

(37) $$\begin{array}{c} \begin{array}{ccc} \hat{s}\left(s\right) & = & -\frac{\xi \omega_{n}^{3}}{g\omega_{ie}\cos L}\cdot \frac{s}{\left(s^{2}+2\xi \omega_{n}s+\omega_{n}^{2}\right)\left(s+\xi \omega_{n}\right)}\cdot \frac{\Delta {A^{\prime }}_{y}}{s}\\ & & +\frac{\xi \omega_{n}^{3}}{\omega_{ie}\cos L}\cdot \frac{1}{\left(s^{2}+2\xi \omega_{n}s+\omega_{n}^{2}\right)\left(s+\xi \omega_{n}\right)}\cdot \frac{{\varepsilon^{\prime }}_{x}+\omega_{ie}\cos L\cdot \sin\gamma }{s} \end{array} \end{array}$$

Similarly, the frequency domain Equation of $\hat{c}$ is:

(38) $$\begin{array}{c} \begin{array}{ccc} \hat{c}\left(s\right) & = & \frac{1}{s}+\frac{\xi \omega_{n}^{3}}{g\omega_{ie}\cos L}\cdot \frac{s}{\left(s^{2}+2\xi \omega_{n}s+\omega_{n}^{2}\right)\left(s+\xi \omega_{n}\right)}\cdot \frac{\Delta {A^{\prime }}_{x}}{s}\\ & & +\frac{\xi \omega_{n}^{3}}{\omega_{ie}\cos L}\cdot \frac{1}{\left(s^{2}+2\xi \omega_{n}s+\omega_{n}^{2}\right)\left(s+\xi \omega_{n}\right)}\cdot \frac{{\varepsilon^{\prime }}_{y}+\omega_{ie}\cos L\cdot \left(\cos\gamma -1\right)}{s} \end{array} \end{array}$$

Equations (37) and (38) show that the azimuthal alignment system of the improved DMCS-based alignment algorithm constructed by Equation (36) is a third-order system. Its characteristic polynomial is:

(39) $$\hat{\Delta }\left(s\right)=\left(s^{2}+2\xi \omega_{n}s+\omega_{n}^{2}\right)\left(s+\xi \omega_{n}\right)$$

If the pole can be configured optimally in the DMCS-based alignment algorithm, its characteristic polynomial:

(40) $$\Delta \left(s\right)=\left(s^{2}+2\xi \omega_{n}s+\omega_{n}^{2}\right){\left(s+\xi \omega_{n}\right)}^{2}$$

Therefore, the azimuth alignment system of the DMCS-based alignment algorithm is a fourth-order system. Due to the lack of a pole in the third-order system, the suppression ability of the external disturbance will be weakened.

In order to ensure the alignment accuracy, the inertia link H(s) is added after $\hat{s}$ and $\hat{c}$, and estimations of sinγ and cosγ are re-established, denoted by $\tilde{s}$ and $\tilde{c}$:

(41) $$\left\{\begin{array}{c} \tilde{s}\left(s\right)=H\left(s\right)\cdot \hat{s}\left(s\right)\\ \tilde{c}\left(s\right)=H\left(s\right)\cdot \hat{c}\left(s\right) \end{array}\right.$$

where:

(42) $$H\left(s\right)=\frac{\xi \omega_{n}}{s+\xi \omega_{n}}$$

According to the final value theorem, when the system is steady, the estimation errors of $\tilde{s}$ and $\tilde{c}$ are:

(43) $$\left\{\begin{array}{c} \delta \left(\sin\gamma \right)=-\frac{{\varepsilon^{\prime }}_{x}}{\omega_{ie}\cos L}\\ \delta \left(\cos\gamma \right)=-\frac{{\varepsilon^{\prime }}_{y}}{\omega_{ie}\cos L} \end{array}\right.$$

**Figure 10:**
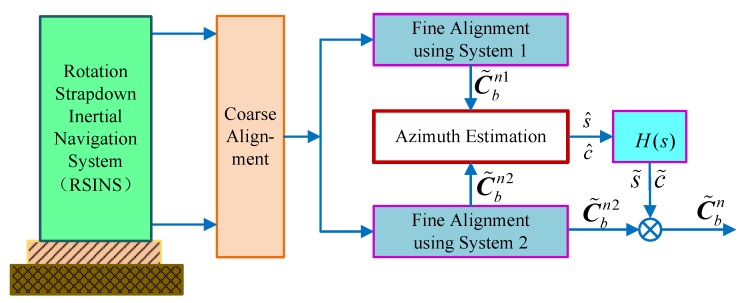
The schematic diagram of improved DMCS-based alignment algorithm.

Therefore, the schematic diagram of the improved DMSC-based alignment algorithm with a large azimuth misalignment angle is shown in Figure 10. From the design principle of this improved alignment algorithm, we can see that the system is linear and that the pole is not affected by the azimuth misalignment angle. Compared with the traditional compass alignment algorithm, the improved DMCS-based alignment algorithm can enhance not only the alignment accuracy, but also the alignment speed when the azimuth alignment angle is large.

## 4. Simulation and Analysis

In order to verify the feasibility of the initial alignment algorithm based on the improved DMCS for the single-axis RSINS in the condition of a large azimuth misalignment angle, the simulations are carried out in this section. As the comparison groups, the initial alignment algorithm based on the traditional compass method and the one based on the traditional DMCS are both simulated here.

### 4.1. Simulation Condition and Settings

**Figure 11:**
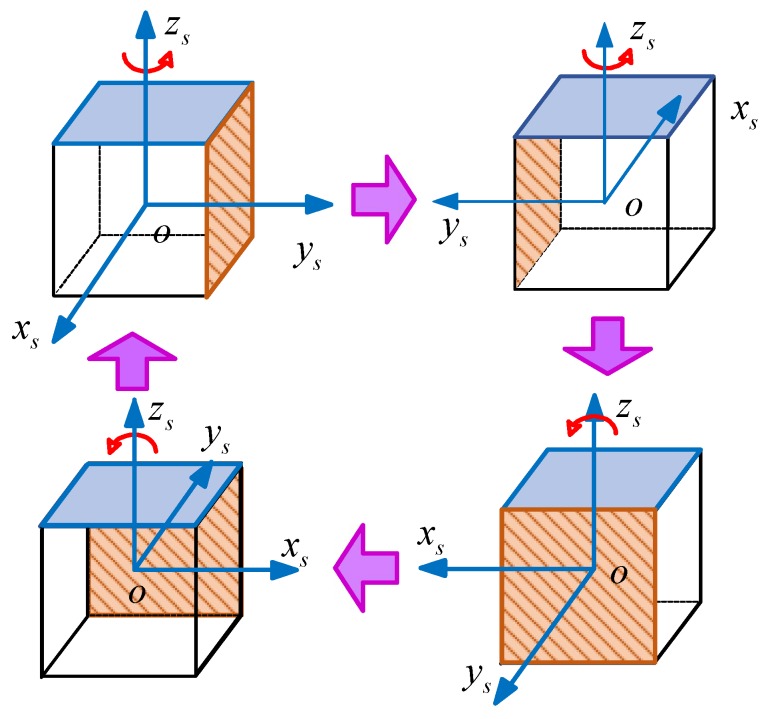
The rotation scheme of the single-axis RSINS.

The simulation model of RSINS is programmed by MATLAB. Taking the literature [27,29,30] and the actual situation of the turntable as references, the simulation conditions and settings are listed as follows:

- Considering the *b*-frame coincides with the *n*-frame, the initial latitude and longitude are set as $45.7796^{\circ }$ and $126.6761^{\circ }$, respectively;
- The accelerometers’ biases are set as $10^{-4}g_{0}$; the gyroscopes’ biases are set as $10^{-2\circ }/h$;
- The random noises of the accelerometer and the gyroscope are set as $5\times 10^{-5}g_{0}$ and $5\times 10^{-3\circ }/h$, respectively;
- Scale factor errors of the accelerometer and the gyroscope are set as 10 ppm;
- The natural oscillation frequency $\omega_{n}$ is set to 0.02 Hz;
- The initial horizontal misalignment angles are set as $0.2^{\circ }$, and the azimuth initial misalignment angles are set as $125^{\circ }$ and $-170^{\circ }$, respectively.
- The rotation scheme is shown as Figure 11, and the rotating rate is $6^{\circ }/s$.

### 4.2. Simulation Results

#### 4.2.1. Initial Alignment Results

**Figure 12:**
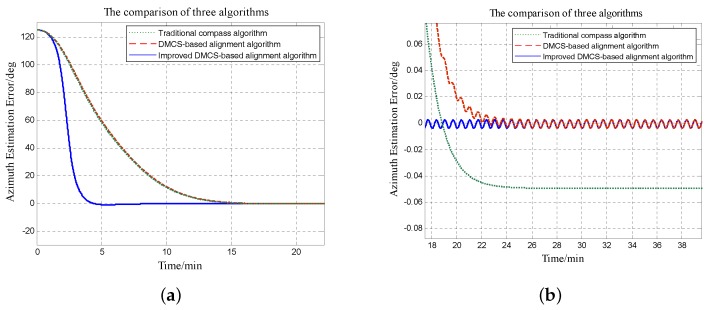
Error curves of the azimuth misalignment angle by utilizing three different initial alignment methods when the initial azimuth misalignment angle is $125^{\circ }$. The green dotted line denotes the initial alignment algorithm based on the traditional compass method; the red dashed line denotes the error curve with the initial alignment algorithm based on the DMCS method; the blue solid line denotes the error curve with the improved DMCS-based initial alignment algorithm. (**a**) Errors of the azimuth misalignment angle. (**b**) Magnification of azimuth misalignment angle errors.

**Figure 13:**
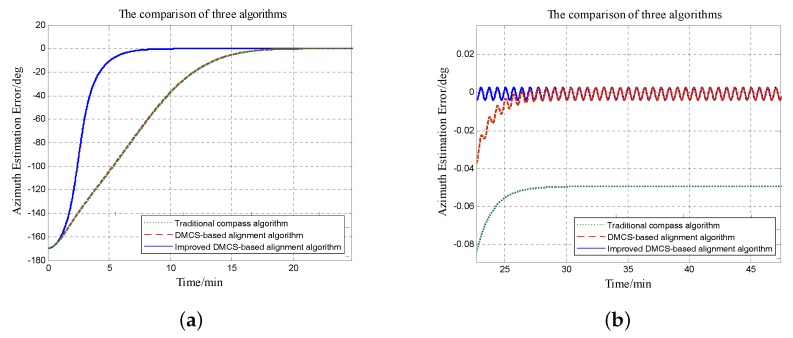
Error curves of the azimuth misalignment angle by utilizing three different initial alignment methods when the initial azimuth misalignment angle is $-170^{\circ }$. The green dotted line denotes the initial alignment algorithm based on the traditional compass method; the red dashed line denotes the error curve with the initial alignment algorithm based on the DMCS method; the blue solid line denotes the error curve with the improved DMCS-based initial alignment algorithm. (**a**) Errors of the azimuth misalignment angle. (**b**) Magnification of azimuth misalignment angle errors.

Since the azimuth misalignment angle has more influence on system performance and the errors of horizontal misalignment angles are not much different, we focus on errors of azimuth misalignment angles with different initial alignment algorithms here. Figure 12 and Figure 13 show error curves under the circumstances that the initial azimuth angle error is $125^{\circ }$ and $-170^{\circ }$, respectively. The right figures are the local magnification of the left figures.

From Figure 12, it is obvious that the convergence time is about 25 min and the steady error is about $0.05^{\circ }$ by utilizing the initial alignment algorithm based on the traditional compass method; the convergence time is still nearly 25 min while the steady error is decreased to $0.005^{\circ }$ with the initial alignment algorithm based on the DMCS method; the convergence time is about 10 min and the steady error is $0.005^{\circ }$ when utilizing the improved DMCS-based initial alignment algorithm.

Figure 13 shows the error curves of azimuth misalignment angles when the initial azimuth misalignment angle is $-170^{\circ }$. The convergence time is about 30 min, and the steady error is $-0.05^{\circ }$ with the initial alignment algorithm based on the traditional compass method; the convergence time is still 30 min and the steady error is about $0.005^{\circ }$ when utilizing the initial alignment algorithm based on the DMCS method; the convergence time is reduced to 10 min and the steady error is about $0.005^{\circ }$ by utilizing the improved DMCS-based initial alignment algorithm.

It is obvious that when the azimuth misalignment angle is large, the initial alignment algorithm with the DMCS method and the one with the improved DMCS method can obtain the same final alignment results, that means the accuracy of these two algorithms is nearly the same; however, the convergence speed of the improved DMCS-based alignment algorithm is much faster.

#### 4.2.2. Pure Inertial Navigation Results

**Figure 14:**
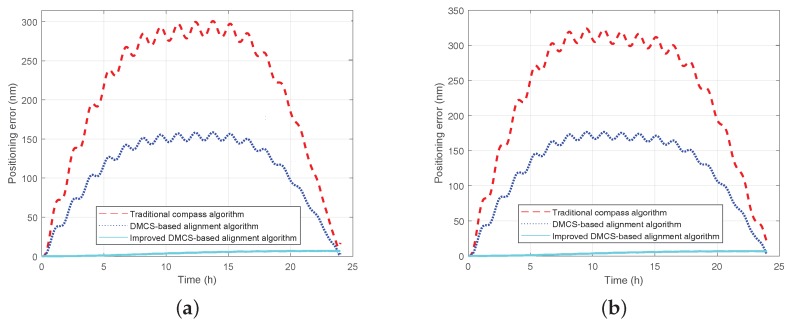
Positioning errors by utilizing the three initial alignment algorithms. The red dashed line denotes the positioning error curve by utilizing the traditional compass algorithm under the static base; the blue dotted line denotes the positioning error by utilizing the DMCS alignment algorithm under the single-axis rotating base; the light blue solid line denotes the positioning error by utilizing the improved DMCS-based alignment algorithm under the rotating base. (**a**) Positioning error when the initial heading error is $125^{\circ }$. (**b**) Positioning error when the initial heading error is $-170^{\circ }$.

It is known from the above simulation results that the improved DMCS-based initial alignment algorithm has better performance both in initial alignment time and accuracy compared with the other two alignment algorithms. The performance of pure inertial navigation will be tested here. It is assumed that the RSINS turns into the the pure inertial navigation status after the initial alignment status, which lasts 15 min. The simulation lasts 24 h, and the positioning error curves are shown in Figure 14. In the figures, the red dashed line denotes the positioning error curve by utilizing the initial alignment algorithm based on the traditional compass method under the static base (denoted as the ‘traditional compass algorithm’); the blue dotted line denotes the positioning error by utilizing the initial alignment algorithm based on the DMCS method under the single-axis rotating base (denoted as the ‘DMCS-based alignment algorithm’); the light blue solid line denotes the positioning error by utilizing the improved DMCS-based initial alignment algorithm under the rotating base (denoted as the ‘improved DMCS-based alignment algorithm’).

From Figure 14, we can see that:

(1) When the initial heading error is $125^{\circ }$, the maximum positioning error can reach 300 nm when utilizing the traditional initial alignment algorithm under the static base; the maximum positioning error is about 160 nm when utilizing the initial alignment algorithm based on the DMCS method under the rotating base; the maximum positioning error is only 6 nm when utilizing the improved DMCS-based initial alignment algorithm under the rotating base.
(2) When the initial heading error is $-170^{\circ }$, the maximum positioning error can reach 325 nm when utilizing the traditional initial alignment algorithm based on traditional compass method under the static base; the maximum positioning error is about 177 nm when utilizing the initial alignment algorithm based on the DMCS method under the rotating base; the maximum positioning error is about 7 nm when utilizing the improved DMCS-based initial alignment algorithm under the rotating base.
(3) Comparing the red line and the blue line, we can know that the RMT could improve the performance of the inertial navigation system due to the inertial sensors’ mitigation.
(4) Comparing the blue line and the light blue line, the results show that the initial alignment accuracy has a large effect on the performance of pure inertial navigation, and the improved DMCS-based alignment algorithm can achieve better navigation performance with little impact on the initial azimuth misalignment angle.

## 5. Real Test and Analysis

### 5.1. Test Environment Establishment

**Figure 15:**
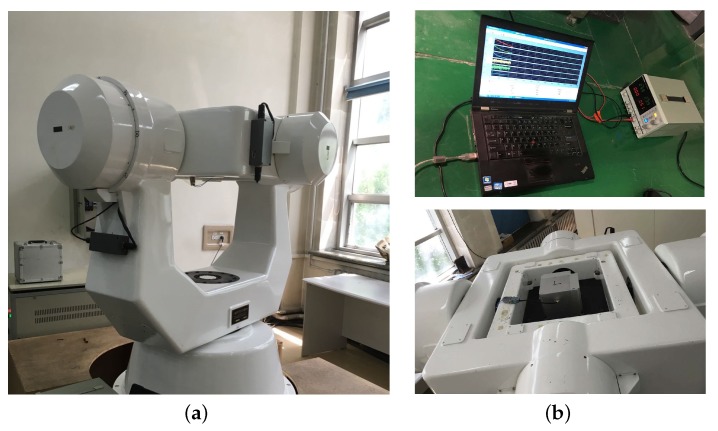
Test environment establishment. (**a**) Three-axis test turntable. (**b**) Test IMU and data collection equipments.

The test environment was established at the Inertial Test Center, Harbin Institute of Technology. The test equipment included the three-axis turntable, the tested IMU, the power supply unit and the data collection unit. The whole test environment is shown as Figure 15. The performance of the tested IMU is illustrated in Table 1, and the raw data were collected by utilizing the data collection unit and processed off-line.

The test scheme was designed as follows:

Test 1: The turntable was kept horizontal and stable, and its initial heading was $45^{\circ }$. The turntable was controlled to rotate $125^{\circ }$ clockwise along the outer-axis after two minutes. Then, the IMU worked under the patterns of single-axis rotation. The whole test lasted 20 h.
Test 2: The turntable was kept horizontal and stable, and its initial heading was $45^{\circ }$. The turntable was controlled to rotate $170^{\circ }$ anti-clockwise along the outer-axis after two minutes. Then, the IMU worked under the patterns of single-axis rotation. The whole test lasted 20 h.
Test 3: The turntable was kept horizontal and stable, and its initial heading was $45^{\circ }$. The turntable was controlled to rotate $125^{\circ }$ degrees clockwise along the outer-axis after two minutes. Then, the IMU kept stable. The whole test lasted 20 h.
Test 4: The turntable was kept horizontal and stable, and its initial heading was $45^{\circ }$. The turntable was controlled to rotate $170^{\circ }$ degrees anti-clockwise along the outer-axis after two minutes. Then, the IMU kept stable. The whole test lasted 20 h.

### 5.2. Results and Analysis

In order to verify the effectiveness of the novel initial algorithms, the initial attitude and positioning errors were calculated by utilizing different algorithms. The traditional compass alignment algorithm and the DMCS-based alignment algorithm were taken as the contrast algorithms. In this section, Test 1 and Test 2 were analyzed by using the DMCS-based alignment algorithm and the improved DMCS-based alignment algorithm, while Test 3 and Test 4 were analyzed by using the traditional compass alignment algorithm. Test 1 and Test 2 gave different initial misalignment angles, to verify the algorithm’s repeatability and stability. From the comparison of these three algorithm, the effectiveness and superiority can be fully validated. Table 2 and Table 3 show the results of 15-min initial alignment and 25-min initial alignment, respectively.

**Figure 16:**
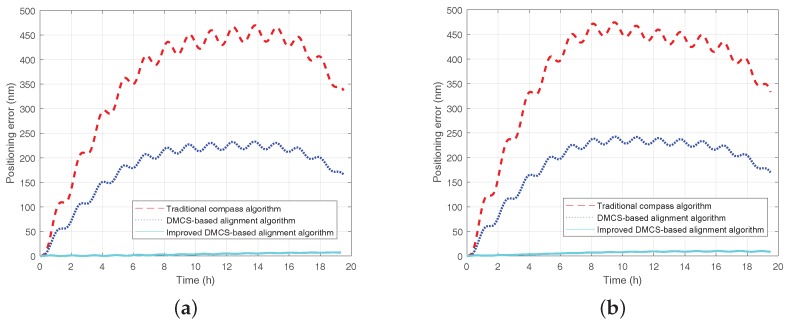
Positioning error curve when the initial alignment lasts 15 min. The red dashed line denotes the positioning error curve by utilizing the traditional compass algorithm under the static base; the blue dotted line denotes the positioning error by utilizing the DMCS alignment algorithm under the single-axis rotating base; the light blue solid line denotes the positioning error by utilizing the improved DMCS-based alignment algorithm under the rotating base. (**a**) Positioning error when the initial heading error is $125^{\circ }$. (**b**) Positioning error when the initial heading error is $-170^{\circ }$.

**Figure 17:**
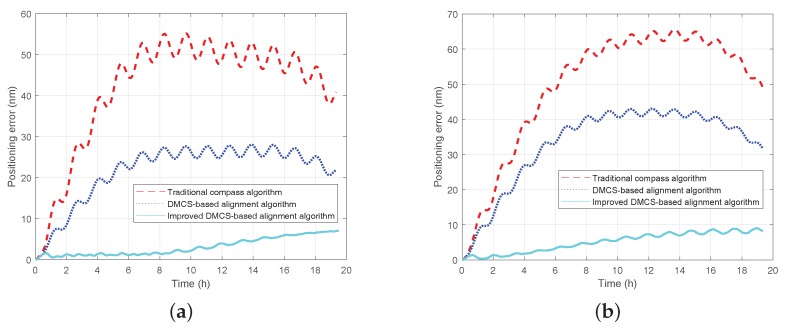
Positioning error curve when the initial alignment lasts 25 min. The red dashed line denotes the positioning error curve by utilizing the traditional compass algorithm under the static base; the blue dotted line denotes the positioning error by utilizing the DMCS alignment algorithm under the single-axis rotating base; the light blue solid line denotes the positioning error by utilizing the improved DMCS-based alignment algorithm under the rotating base. (**a**) Positioning error when the initial heading error is $125^{\circ }$. (**b**) Positioning error when the initial heading error is $-170^{\circ }$.

Since the three-axis turntable is not calibrated with true north, there is a fixed bias in the alignment result. Therefore, in order to verify the accuracy of the initial alignment, pure inertial navigation is operated based on the initial alignment result of 15 min and 25 min, respectively. The positioning error curves are shown as Figure 16 and Figure 17. The positioning error is summarized in Table 4.

We can see from Figure 16, Figure 17 and Table 4 that:

- The RMT could suppress the impact of the inertial sensors’ error on the inertial navigation system effectively to enhance the performance of the inertial navigation system;
- The performance of the initial alignment determines the accuracy of the inertial navigation system, and the initial alignment of high accuracy is the precondition and guarantee for high accuracy inertial navigation;
- The initial alignment algorithm based on DMCS method has better performance and a faster convergence rate compared with the initial alignment algorithm based on compass method; what is more, the improved DMCS-based initial alignment algorithm can obtain the best performance when the azimuth misalignment angle is large.

## 6. Conclusions

In order to improve the applicability of the RSINS for submarines, a novel initial alignment algorithm is proposed in this manuscript. Considering the alignment accuracy of the traditional compass alignment method, we give a novel alignment algorithm based on the DMCS method; on that basis, taking the large azimuth misalignment angle into account, an improved DMCS-based alignment algorithm is proposed, suppressing the influence of the large misalignment angle on the initial alignment. Results of the simulations and experiments showed that this improved DMCS-based alignment algorithm is superior not only with respect to the accuracy, but also the convergence speed compared to the traditional algorithm when the misalignment angle is large, significantly enhancing RSINS’s performance.

References

## Figures and Tables

**Table 1:** Performance of the tested IMU.

	Parameters	Indexes
Gyroscope	Bias Instability	$0.01^{\circ }/h$
	Bias Repeatability	$0.01^{\circ }/h$
	Random Walk Coefficient	$0.001^{\circ }/\sqrt{h}$
	Nonlinearity of Scale Factor	20 ppm
	Dynamic Range	$\pm 300^{\circ }/s$
Accelerometer	Bias Instability	$10^{-4}g_{0}$
	Random Noise	$5\times 10^{-5}g_{0}$
	Nonlinearity of Scale Factor	10 ppm
	Dynamic Range	$\pm 20g_{0}$

**Table 2:** Initial alignment results after 15 min.

		Pitch (${}^{\circ }$)	Roll (${}^{\circ }$)	Yaw (${}^{\circ }$)
Test 3	Traditional compass algorithm	0.072	0.092	176.785
Test 1	DMCS-based alignment algorithm	0.018	0.027	173.335
	Improved DMCS-based alignment algorithm	0.005	0.014	169.713
Test 4	Traditional compass algorithm	−0.084	0.098	226.515
Test 2	DMCS-based alignment algorithm	−0.039	0.032	230.675
	Improved DMCS-based alignment algorithm	0.006	0.014	234.716

**Table 3:** Initial alignment results after 25 min.

		Pitch (${}^{\circ }$)	Roll (${}^{\circ }$)	Yaw (${}^{\circ }$)
Test 3	Traditional compass algorithm	0.020	0.034	170.575
Test 1	DMCS-based alignment algorithm	0.008	0.015	170.155
	Improved DMCS-based alignment algorithm	0.004	0.011	169.672
Test 4	Traditional compass algorithm	−0.029	0.040	233.675
Test 2	DMCS-based alignment algorithm	−0.015	0.022	234.025
	Improved DMCS-based alignment algorithm	0.004	0.012	234.674

**Table 4:** Comparison of positioning errors.

			Positioning Error (nm)
15 min	Test 3	Traditional compass algorithm	469.69
	Test 1	DMCS-based alignment algorithm	233.13
		Improved DMCS-based alignment algorithm	7.4
	Test 4	Traditional compass algorithm	469.69
	Test 2	DMCS-based alignment algorithm	233.13
		Improved DMCS-based alignment algorithm	7.4
25 min	Test 3	Traditional compass algorithm	55.17
	Test 1	DMCS-based alignment algorithm	28.05
		Improved DMCS-based alignment algorithm	7.05
	Test 4	Traditional compass algorithm	65.52
	Test 2	DMCS-based alignment algorithm	43.04
		Improved DMCS-based alignment algorithm	9.03
